# Embryonic Cerebrospinal Fluid Increases Neurogenic Activity in the Brain Ventricular-Subventricular Zone of Adult Mice

**DOI:** 10.3389/fnana.2017.00124

**Published:** 2017-12-19

**Authors:** Maria I. Alonso, Francisco Lamus, Estela Carnicero, Jose A. Moro, Anibal de la Mano, Jose M. F. Fernández, Mary E. Desmond, Angel Gato

**Affiliations:** ^1^Departamento de Anatomía y Radiología, Facultad de Medicina, Universidad de Valladolid, Valladolid, Spain; ^2^Laboratorio de Desarrollo y Teratología del Sistema Nervioso, Instituto de Neurociencias de Castilla y León (INCYL), Universidad de Valladolid, Valladolid, Spain; ^3^Departamento de Biología Celular, Histología y Farmacología, Facultad de Medicina, Universidad de Valladolid, Valladolid, Spain; ^4^Department of Biology, Villanova University, Villanova, PA, United States

**Keywords:** embryonic cerebrospinal fluid (CSF), brain development, ventricular-subventricular zone (SVZ), neural stem cells, neurogenesis, stem-cells, brain

## Abstract

Neurogenesis is a very intensive process during early embryonic brain development, becoming dramatically restricted in the adult brain in terms of extension and intensity. We have previously demonstrated the key role of embryonic cerebrospinal fluid (CSF) in developing brain neurogenic activity. We also showed that cultured adult brain neural stem cells (NSCs) remain competent when responding to the neurogenic influence of embryonic CSF. However, adult CSF loses its neurogenic inductive properties. Here, by means of an organotypic culture of adult mouse brain sections, we show that local administration of embryonic CSF in the subventricular zone (SVZ) niche is able to trigger a neurogenic program in NSCs. This leads to a significant increase in the number of non-differentiated NSCs, and also in the number of new neurons which show normal migration, differentiation and maturation. These new data reveal that embryonic CSF activates adult brain NSCs, supporting the previous idea that it contains key instructive components which could be useful in adult brain neuroregenerative strategies.

## Introduction

Neural stem cells (NSCs) in the adult mammalian brain are regarded as a useful tool to regenerate neurons in pathological conditions such as neurodegenerative diseases or acute brain injury. Neurogenic potential has been demonstrated in only two specific areas of the normal mammal brain in adults, namely, the subventricular zone (SVZ) of the lateral ventricle (LV) and the subgranular layer of the hippocampal dentate gyrus (DG). In mammals, both locations show neurogenic activity throughout life (Alvarez-Buylla and Lim, 2004; Lathia et al., 2007; Kazanis et al., 2008). This neurogenic activity is not so intense in normal physiological conditions, but several studies, have shown the existence of a non-developed reserve of neurogenic activity in the SVZ, which is made evident in cerebral tissue damage (Yamashita et al., 2006; Kaneko and Sawamoto, 2009; Kernie and Parent, 2010; Kaneko et al., 2011; Chang et al., 2016).

In the last few years, the study of the sequence of cellular events from NSCs to mature neurons, has led to a functional concept, the “cellular niche” (Alvarez-Buylla and Garcia-Verdugo, 2002; Merkle and Alvarez-Buylla, 2006; Quiñones-Hinojosa et al., 2006), which includes not only the NSCs and their progeny, but also other mature cells in the surrounding area (neurons, glia and ependimocytes), the extracellular matrix, blood vessels and cerebrospinal fluid (CSF), which is in contact with this complex cellular structure. The complex microenvironment of diffusible signals and intercellular influences seems to be responsible for the regulation of neurogenic activity in the adult brain niche (Lathia et al., 2007; Zhao et al., 2008; Ming and Song, 2011; Lim and Alvarez-Buylla, 2014).

E-CSF is a complex fluid involved in several brain developmental mechanisms which include a key role in neurogenesis (Gato et al., 2005; Parada et al., 2005b; Martin et al., 2009; Desmond et al., 2014; Chau et al., 2015). This ability seems to be based on the presence of several molecules of a high biological value (Gato and Desmond, 2009). Many of these molecules are proteins, as has been shown mainly by proteomic analysis (Gato et al., 2004; Parada et al., 2005a, 2006; Zappaterra et al., 2007; Chau et al., 2015). Several growth and transcriptional factors have been identified in CSF as active components; these include FGF2, IGF1 and EGF which has been witnessed in the regulation of the mitotic activity of NSCs (Miyan et al., 2003; Martín et al., 2006; Lathia et al., 2007; Zappaterra and Lehtinen, 2012), and the “retinol binding protein”, which regulate the intake of Retinol, the Retinoic Acid precursor, in RALDH positive cells responsible for Retinoic Acid synthesis (Parada et al., 2008; Alonso et al., 2011, 2014; Chang et al., 2016).

NSCs have been identified as neuroepithelial cells early in development, radial glia in the fetal period and specific astrocytes in the adult brain in mammals. They have been considered a unique cellular lineage which evolves ontogenically (Kriegstein and Alvarez-Buylla, 2009), whilst preserving their potential of self-renewal and pluripotent differentiation. However, they exhibit decreasing neurogenic activity with age in accordance with ontogenical modifications in niche composition and signaling. Several research studies (Alvarez-Buylla and Garcia-Verdugo, 2002; Gato et al., 2005; Lehtinen and Walsh, 2011) reveal the existence of a specific spatial relation of the NSCs niche with the ventricular system and CSF. This direct contact is particularly evident in neuroepithelial and radial glia cells, but has also been described in adult brain astrocytic NSCs of the SVZ (Merkle and Alvarez-Buylla, 2006). The influence of CSF on NSCs behavior has been highlighted in the last few years both during development (Miyan et al., 2003; Gato et al., 2005; Gato and Desmond, 2009), and also in the adult brain (Lehtinen and Walsh, 2011; Lehtinen et al., 2011; Zappaterra and Lehtinen, 2012). In fact, CSF is considered to be a main component of niche signaling (Lathia et al., 2007).

However, studies have described different effects of CSF upon NSCs during development (induction of precursor replication and neurogenesis, Gato et al., 2005; Alonso et al., 2011; Chau et al., 2015), compared with those encountered during the adult period (role in migratory guidance, Sawamoto et al. (2006), or the induction of gliogenesis, Buddensiek et al., 2010). The contradictory role of CSF throughout life can be explained in terms of ontogenic changes in its composition (Carnicero et al., 2013), which range between Embryonic CSF (E-CSF), with a high degree of neurogenic power, to Adult CSF (A-CSF) with low neurogenic potential; however, it can also be explained through the differential competence of adult and embryonic NSCs. In this regard, previous studies have shown that, at earliest stages of development, E-CSF has a complex composition with several molecules such as growth factors and morphogens (Kazanis et al., 2008; Pathania et al., 2010; Lun et al., 2015) that function as a cocktail of signals able to induce survival, replication and neuronal differentiation in developing brain stem cells (Gato et al., 2005; Martin et al., 2009).

Taking into consideration these concepts together with our preliminary study showing that E-CSF is able to induce the initial steps of neuronal differentiation on SVZ NSCs cultured “*in vitro*” outside the niche (Carnicero et al., 2013), we assess the potential of E-CSF on the entire behavior of adult NSCs.

## Materials and Methods

Our experimental approach was based on an organotypic tissue culture of adult mouse brain sections (Schommer et al., 2017) from the SVZ, with local administration of CSF soaked latex micro-beads (Gato et al., 2005).

### Obtaining Cerebrospinal Fluid

E-CSF was micro-aspirated from the mesencephalic cavity of 12.5-day-old mouse embryos (Swiss-Webster strain), as previously described (Martin et al., 2009).

A-CSF was obtained from 3- to 4-month-old mice of both sexes, by micropuncture of the “cisterna magna”, in accordance with the technique of Liu and Duff (2008).

To minimize protein degradation, CSF samples were kept at 4°C during handling and then lyophylized and frozen at −40°C until use.

### Organotypic Culture of Sections Containing Lateral Ventricle SVZ

**Figure 1:**
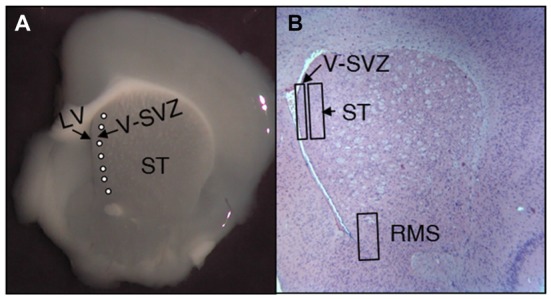
Representative images of adult mice brain slices at the subventricular zone (SVZ) level. **(A)** Vibratome coronal slices showing the striatum (ST), the lateral ventricle (LV) and the SVZ; latex micro-beads soaked in PBS or CSF were implanted immediately behind the SVZ (white circles). **(B)** Coronal histological section stained with hemeatoxylin-eosin at the same level as **(A)**. The image shows the location of the predetermined areas to study the number and characteristics of neural stem cells (NSCs) for the neurogenic niche (SVZ), the neighbor striatum (ST) and the rostral migratory stream (RMS).

Organotypic cultures were performed according to current protocols (Stoppini et al., 1991; Schommer et al., 2017). Animals were housed and handled in ways that minimize pain and discomfort, in accordance with Spanish animal welfare regulations (RD53/2013) and in agreement with the European community Council Directive (2010/63/EU). Adult mice brains from both sexes were obtained by surgery under deep anesthesia, following Spanish animal care legislation (approved by Comité de Ética en Experimentació Bienestar Animal-CEEBA–University of Valladolid). Authorization for the experiments was granted by the CEEBA-University of Valladolid and the Consejeria de Agricultura de la JCYL) and the experiments were performed under the surveillance of the animal welfare officer responsible for the Valladolid University. Adult mice brains from both sexes were used. Upon removal, each brain hemisphere was immersed in sterile saline at 4°C, then placed in 3% liquid agar and stored for 1.5 h at 4°C. Coronal 300-μm-thick sections from the SVZ were obtained with a Vibratome, and carefully placed onto a Millipore filter paper (0.8 μm pore diameter). A total of 5–6, latex micro-beads (50–100 μm diameter Sigma SD-91) were implanted in each brain slice with micro-forceps, close to the ventricular surface, as is shown in Figure 1. Latex micro-beads were previously soaked for 24 h at 4°C either in the culture medium, in E-CSF or A-CSF. Finally, brain sections were covered with a collagen layer (collagen 1% in DMEM-Fetal Bovine Serum 8:1:1). Brain sections were then cultured in a floating system, as previously reported (Gato et al., 2005). We used DMEM supplemented culture medium with 1% Penicillin/Streptomicyn +25% of horse serum + 6 mg/ml Glucose +25% HBSS, at 37°C and in a 5% CO_2_ atmosphere for 6 days. The culture medium was also supplemented with BrdU (10 μM) to detect cellular replication. Following culture, the samples were fixed in Carnoy’s solution for 1 h, embedded in paraffin and sectioned at 8 μm for immunohistochemistry. We performed a total of five different cultures, generating 30 SVZ cultured brain slices for experimental condition (Control, E-CSF and A-CSF).

### Immunostaining

To evaluate NSCs behavior, histological sections obtained from the *in vitro* cultured SVZ brain slices were used for single or double immunolabeling procedures in order to evaluate NSCs replication, migration and differentiation. For this purpose, we have chosen some of the more representative antibodies in order to clarify the influence of E-CSF in SVZ niche dynamics.

The BrdU added to the culture media, was incorporated into the nuclei of DNA synthesizing cells, which in the SVZ correspond with NSCs. This procedure allows the identification of the stem cells and their offspring for a couple of cell divisions; consequently, BrdU-immunolabeling was used to evaluate the replication rate and also as a cellular lineage tracer in our SVZ culture system to follow the presence of NSCs in the striatum and in the rostral migratory stream (RMS).

To assess whether the NSCs population brings about an increase in SVZ neurogenesis, we evaluated the presence of BrdU co-localized with Sox2, a marker for NSCs in an undifferentiated state; Neurod1, a transcription factor widely used as an early stage neuron commitment marker; βIII-Tubulin (Tuj-1) a widespread young neurons marker; Doublecortin, a specific marker for migratory NSCs and Calretinin, a differentiated neuronal marker (Perez-Asensio et al., 2013; Kaur et al., 2015; Ochi et al., 2016).

Immunolabeling was performed following standard procedures and the antibodies used for immunostaining were as follows: **Anti BrdU** (1/50 dilution, Dako, Ref. M7240); **Anti Sox2 (D-17)** (1/50 dilution, Santa Cruz Biotechnology, Ref.sc-17319); **Anti Neurod1** (1/200 dilution, Sigma, Ref. T2200); **Anti β-III-Tubulin (Tuj 1)** (1/20 dilution SIGMA, Ref. T2200); **Anti Calretinin** (1:200 dilution, Millipore AB5054); and **Anti Doublecortin (DCX)** (1/20 dilution, Abcam ab18723). Secondary antibody for Anti Sox2 was Antigoat Ig G-Alexa 594 (1/1000 dilution Invitrogen, Ref. A110 58). Secondary antibody for the rest was Antimouse Ig G-Alexa 488 (Invitrogen, Ref. 10680), 1/1000 dilution.

Immunolabeled cells were photographed with a Leica TCS SPE confocal laser microscope.

### Quantification and Statistical Analysis

To quantify the results, we randomly selected 20 laser confocal brain images taken from 10 different animals (*n* = 10) for each experimental condition (Control, E-CSF and occasionally A-CSF) and immunolabeling type. All images (which had a 0.0269 mm^2^ area) were carefully obtained in the selected zones: SVZ, striatum (ST) and RMS defined in Figure 1B. The total number of BrdU or double immunolabeled cells in each case was counted and plotted in the graph bars as mean ± standard deviation. Statistical analyses of data were conducted by one-way ANOVA analysis of variance followed by a *post hoc* Bonferroni test, or alternatively we used the two-tailed Student’s *t*-test. In both cases the significance threshold was set as *p* ≤ 0.05 or *p* ≤ 0.001.

## Results

In this study, we evaluated the effect of E-CSF on the behavior of SVZ NSCs in the adult mouse brain, taking into consideration cellular replication, neuronal differentiation, migration and maturation.

### E-CSF Expands the Neural Precursor Cell Population in the SVZ

In order to evaluate the effect of E-CSF on SVZ NSCs replication, we focused our attention on two different locations; the first was the tissue underlying the ependymal layer lining the anterior horn of the LV (Figures 1A,B, SVZ); the second was the striatal tissue close to the SVZ (Figures 1A,B, ST). Both areas showed BrdU positive nuclei in control and experimental conditions and were close to the latex micro-bead implants (Figure 1A). In our experience, E-CSF soaked latex micro-bead implants are able to influence the surrounding area for several days (Gato et al., 2005); moreover, as we showed in Figure 1A, latex micro-bead implants were located very close to the brain ventricle, allowing E-CSF diffusible signals to interact with the ventricular surface.

**Figure 2:**
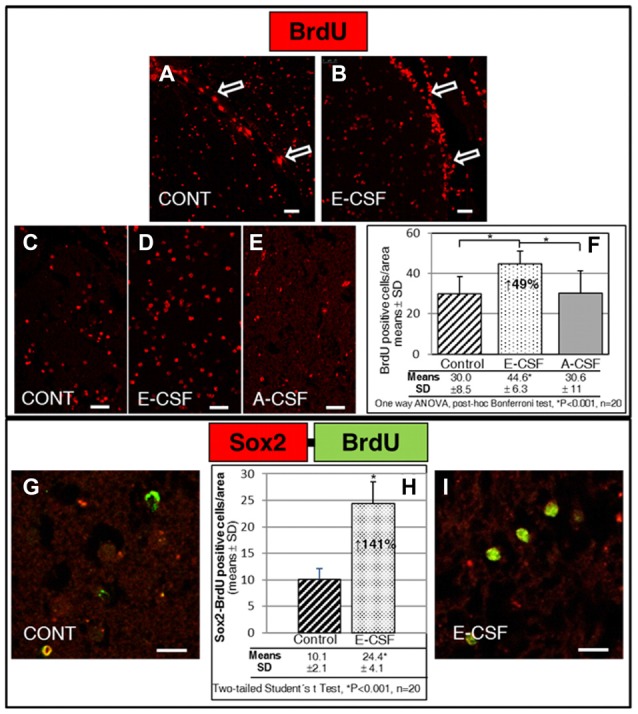
Effect of Embryonic CSF (E-CSF) on mitotic activity in SVZ niche NSCs, monitored by nuclear incorporation of BrdU (red). Confocal photomicrographs (**A,B** show the SVZ of “*in vitro*” cultured adult mice brain slices (see Figure 1B). Note the substantial increase in NSCs mitotic activity under the ventricular surface (white arrows) induced by E-CSF **(B)**, compared with the controls **(A)**. Confocal photomicrographs **(C–E)** show the Striatum area of “*in vitro*” cultured adult mice brain slices close to the SVZ (ST in Figure 1B). Quantification of BrdU positive nuclei in Control **(C)**, E-CSF **(D)** and A-CSF **(E)** treated brain slices was plotted in graph bars **(F)** and expressed as means ± SD (*n* = 20); the significant threshold was set at *p* ≤ 0.001 (*) according to the one-way ANOVA, *post hoc* Bonferroni test. The results reveal a statistically significant increase (49%) in the number of BrdU positive NSCs in CSF-E treated brain slices with respect to the controls and A-CSF treated slices; this suggests a specific activation by E-CSF of mitotic activity in NSCs of the SVZ niche. Confocal photomicrographs **(G,I)** correspond to the striatum area of “*in vitro*” cultured adult mice brain slices, close to SVZ (ST in Figure 1B). Images show double immunolabeling with antiBrdU (green) and antiSox2 (red) antibody; co-localization of both antibodies was interpreted as NSCs dividing (BrdU positive) but not differentiating (Sox2 positive). Quantification of co-labeled BrdU and Sox2 NSCs in Control **(G)** and E-CSF treated brain slices **(I)** was plotted in graph bars **(H)** and expressed as means ± SD (*n* = 20); the significant threshold was set at *p* ≤ 0.001 (*) according to the two-tailed Student’s *t*-test. The results reveal a statistically significant increase (141%) in the number of NSCs that undergo replication but remain undifferentiated in CSF-E treated brain slices, in comparison with the controls. Scale bar: 25 μm **(A–E)** and 10 μm **(G,I)**.

The SVZ in the control sections (Figure 2A) showed the presence of several BrdU positive nuclei directly behind the ventricular surface; however, these positive nuclei were frequently away from each other and appeared as a discontinuous line. Meanwhile, the SVZ in the E-CSF treated sections (Figure 2B) revealed several areas underlying the ventricular surface with an evident increase in the number of BrdU positive nuclei with respect to the control samples, which occasionally formed a continuous cord with several layers of nuclei, indicating an increase in the SVZ replicative activity of NSCs.

Given that highly proliferative NSCs were also located in the striatal tissue close to the SVZ, we chose standard areas to quantify the number of BrdU positive nuclei (see Figure 1B, ST). In the control sections we found a discrete and scattered number of BrdU positive nuclei (Figure 2C). The number of positive nuclei was higher (49%) in the E-CSF treated sections (Figure 2D) with respect to the control ones and was statistically significant (Figure 2F). Regarding A-CSF treated sections (Figure 2E), we could not find significant differences with the controls (Figures 2C,F).

In order to clarify this result, we evaluated in the same area (ST) the nuclear co-localization of BrdU and SOX2, which is a molecular marker of undifferentiated NSCs (Khodosevich et al., 2013). As is shown in Figures 2G–I, E-CSF induces a significant increase (141%) in the number of cells which express both (BrdU and Sox2) with respect to the controls.

### E-CSF Also Induces Neurogenesis in SVZ

Although a considerable number of NSCs cultured with E-CSF remained in an undifferentiated state (BrdU positive and Sox2 positive), there were also many cells (BrdU positive and Sox2 negative) in the SVZ (data not shown), which could be those in a more advanced state of differentiation. To assess whether the NSCs amplified population detected in E-CSF treated sections also brings about an increase in SVZ neurogenesis, we evaluated the expression of Neurod1 at an early stage in the neuronal commitment marker and βIII-Tubulin (Tuj-1) as a young neurons marker.

**Figure 3:**
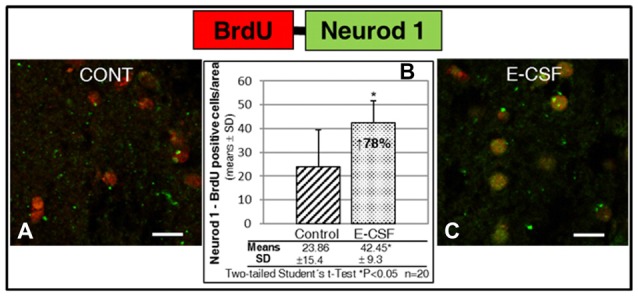
Effect of E-CSF on the number of NSCs with neuronal commitment, monitored by co-expression of BrdU (red) and Neurod1 (green). Confocal photomicrographs show the Striatum area of “*in vitro*” cultured adult mice brain slices close to the SVZ (ST in Figure 1B). Quantification of co-labeled BrdU and Neurod1 NSCs in Control **(A)** and E-CSF treated brain slices **(C)** was plotted in graph bars **(B)** and expressed as means ± SD (*n* = 20); the significant threshold was set at *p* ≤ 0.05 (*) according to the two-tailed Student’s *t*-test. The results reveal a statistically significant increase (78%) in the number of NSCs with neuronal commitment in CSF-E treated brain slices with respect to the controls; this suggests an increase in adult brain neurogenesis activity induced by embryonic CSF. Scale bar: 10 μm **(A,C)**.

As is shown in Figures 3A–C, E-CSF exposure induced in the selected striatum area (Figure 1B) a clear and statistically significant increase (78% with respect to the control ones) in Neurod1 positive cells which also co-express BrdU (as a cellular lineage marker).

**Figure 4:**
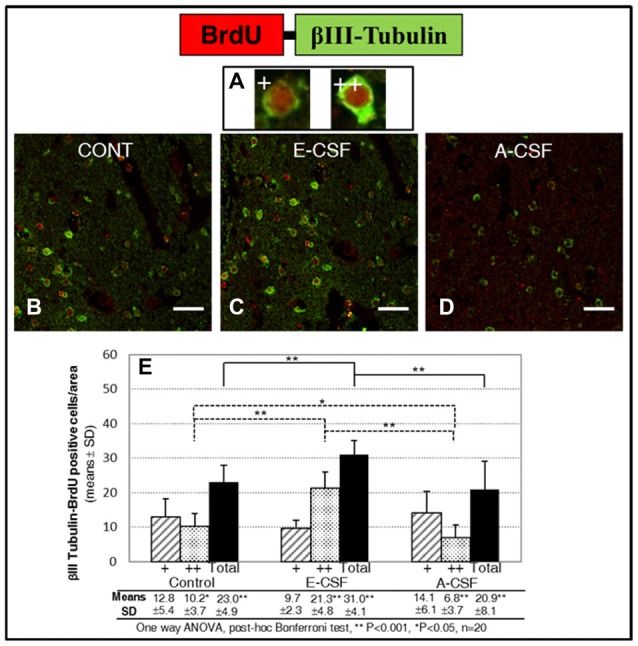
Effect of E-CSF on early neurogenesis, monitored by co-expression of BrdU (red) and Beta III Tubulin (green). Confocal photomicrographs show the Striatum area of “*in vitro*” cultured adult mice brain slices close to the SVZ (ST in Figure 1B). We evaluated individually new neurons showing co-labeling of BrdU with low (**A+**, newborn neurons) and high (**A++**, young neurons) Beta III Tubulin cytoplasmic expression. Quantification of co-labeled NSCs in Control **(B)**, E-CSF **(C)** and A-CSF **(D)** treated brain slices was plotted in graph bars **(E)** and expressed as means ± SD (*n* = 20); the significant threshold was set at *p* ≤ 0.05 (*) or *p* ≤ 0.001 (**) according to the one-way ANOVA, *post hoc* Bonferroni test. The results reveal a statistically significant increase in the total number of new neurons derived from NSCs in E-CSF treated brain slices with respect to the controls (34%). In contrast, A-CSF treated brain slices show a drop (10%) in the total number of new neurons derived from NSCs with respect to the controls. There is also a significant increase in the presence of high-level Beta III Tubulin cytoplasmic expression in E-CSF treated brain slices with respect to the controls (116%) and A-CSF (211%). This suggests that E-CSF significantly increases neurogenesis in SVZ derived NSCs, but also accelerates transition from newborn to young neuronal state. Note that A-CSF does not reproduce this neurogenic activation. Scale bar: 25 μm **(B–D)**.

In order to test whether the increase in neuronal commitment induced by E-CSF leads to an effective and persistent increase in new neurons, we evaluated the number of cells with co-expressed BrdU and βIII-Tubulin in the SVZ. Given the presence of two types of co-labeled cells in the striatum area studied in accordance with the amount of βIII-Tubulin in the cytoplasm and identified as low (Figure 4A+: nuclei partially surrounded by βIII-Tubulin) or high expression (Figure 4A++: nuclei totally surrounded by βIII-Tubulin), we conducted an individual study of both types of new neurons in order to assess changes in the duration of the neurogenetic cycle. As in the NSCs replication study, we included here three experimental conditions: brain slices cultured with latex micro-beads soaked in defined media (Control, Figure 4B), in E-CSF (Figure 4C) and in A-CSF (Figure 4D), in order to ensure the specificity of the neurogenic induction of E-CSF. Results are shown in Figure 4E, which includes the cells with low (+) and high (++) expression of βIII-Tubulin. In basal conditions (control treated with defined media) there was noticeable neurogenic activity in terms of BrdU-βIII-Tubulin co-labeled cells; however, almost 60% showed low βIII-Tubulin expression, which we attributed to early newborn neurons. The sections treated with E-CSF revealed a significant increase in the total number of new neurons (nearly 34%) with respect to the controls, but near to 69% of these cells showed high β3 tubulin expression, which we attributed to young neurons. On the other hand, sections treated with A-CSF displayed a significant decrease (around 10%) in the total number of new neurons, whilst 67.5% of these neurons revealed a low level of βIII-Tubulin expression (newborn neurons) with respect to the control cells.

### E-CSF Induced Neurons Develop a Normal Migratory Pattern

In order to evaluate the normal behavior of the new neurons induced by E-CSF, we studied their migratory capacity by the cytoplasmic expression of Doublecortin (DCX), a specific marker for migratory NSCs, co-labeling with nuclear BrdU expression as a cellular lineage marker. For this purpose we determined the co-labeled cells in areas located at the starting point of the RMS (Figure 1B, RMS), where we expected migratory NSCs to be concentrated in the brain slices culture system.

**Figure 5:**
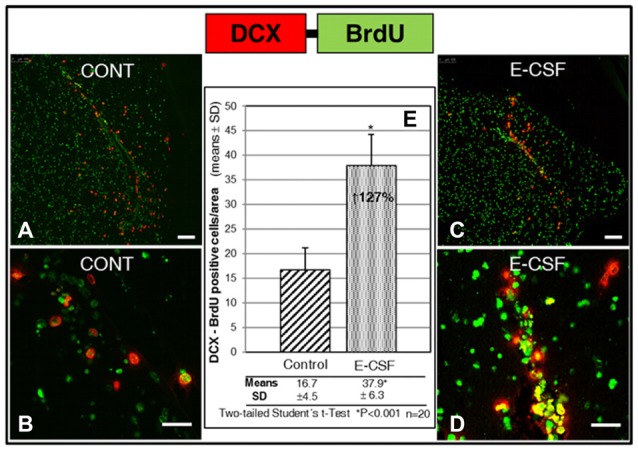
Effect of E-CSF on the number of migratory NSCs, monitored by co-expression of BrdU (green) and Doublecortin (DCX, red). Confocal photomicrographs show the RMS area of “*in vitro*” cultured adult mice brain slices (RMS in Figure 1B). Quantification of co-labeled BrdU and (DCX) NSCs in Control **(A,B)** and E-CSF treated brain slices **(C,D)** was plotted in graph bars **(E)** and expressed as means ± SD (*n* = 20); the significant threshold was set at *p* ≤ 0.001 (*) according to the two-tailed Student’s *t*-test. The results reveal a significant increase (127%) in the number of RMS migratory NSCs in CSF-E treated brain slices with respect to the controls. Scale bar: 100 μm **(A,C)** and 25 μm **(B,D)**.

The results are shown in Figure 5. We found a significant increase of migratory cells congregated in coincidence with the proximal segment of the RMS on E-CSF treated brain sections (Figures 5C,D, images), compared with control brain sections (Figures 5A,B, images). Data were plotted in Figure 5E to show that the increased number of co-labeled cells (127% in E-CSF treated sections with respect to controls was statistically significant.

### Neurogenesis Induced by E-CSF Leads to Neuronal Maturity

The last part of our study sought to answer another relevant question: the evolution to mature neurons of the newly generated neurons in the SVZ niche under E-CSF influence.

**Figure 6:**
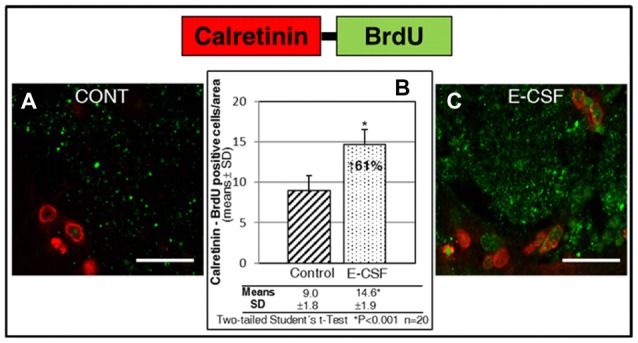
Effect of E-CSF on the number of NSC derived neurons which co-expressed BrdU (green) and Calretinin (mature neuronal marker, red). Confocal photomicrographs show the RMS area of “*in vitro*” cultured adult mice brain slices (RMS in Figure 1B). Quantification of co-labeled BrdU and Calretinin NSCs in Control **(A)** and E-CSF treated brain slices **(C)** was plotted in graph bars **(B)** and expressed as means ± SD (*n* = 20); the significant threshold was set at *p* ≤ 0.001 (*) according to the two-tailed Student’s *t*-test. The results reveal a statistically significant increase (61%) in the number of NSC derived mature neurons in E-CSF treated brain slices with respect to the controls; this suggests that neurogenesis induced by embryonic CSF drives the development of mature neurons. Scale bar: 25 μm **(A,C)**.

Despite not being able to follow the migratory neural precursors to their natural destination, the olfactory bulb, we assume that in our coronal section culture system migratory NSCs stop at the starting point of the RMS, where the maturation of new neurons start. Consequently, here we attempted to locate cells with co-labeling of BrdU (which, despite the decrease in intensity, remained detectable) and Calretinin, a mature neurons marker. As can be seen in Figure 6, the area at the start of the RMS (Figure 1B) shows an accumulation of cells which co-express BrdU-Calretinin; the number of these cells was larger in E-CSF treated sections (Figure 6C) compared with the controls (Figure 6A), representing a statistically significant increase of 61% (Figure 6B).

## Discussion

We previously published (Carnicero et al., 2013) a study showing the direct influence of E-CSF on neurogenic activity in adult brain SVZ NSCs cultured “*in vitro*”; this previous study only showed that E-CSF is able to induce the initial steps of neuronal differentiation in SVZ NSCs cultured “*in vitro*” (outside the niche). This is the first report of the influence of E-CSF in organotypic cultures of the SVZ neurogenic niche; moreover, here we show the influence of E-CSF on the main steps of the physiological behavior of SVZ NSCs including expansion of the un-differentiated NSCs population and also the increase of effective neuronal differentiation, migration and final neuronal maturation.

Our results, based on BrdU incorporation of mitotically active SVZ NSCs, show that E-CSF induces a large number of proliferative NSCs, many of which remain in an un-differentiated neural precursor; consequently, E-CSF seems to be involved in the self-renovation of the SVZ NSCs population. We also show that the mitogenic effect of CSF on NSCs is specific for the E-CSF, suggesting ontogenic changes in SVZ NSCs regulatory mechanims. The presence of BrdU positive nuclei with Sox2 negative label in the SVZ and adjacent ST reflects NSCs which underwent proliferation but are not necessarily in the transitory amplifying state, since nuclear BrdU is also detectable at the stages of differentiation and maturation. Therefore, we cannot assign the overall increase in BrdU positive nuclei induced by E-CSF exclusively to an increase in the NSCs (undifferentiated) population. Our results have also shown that, under E-CSF influence, a significant number of proliferative SVZ NSCs initiate a process of neurogenesis (Neurod1 or βIII-Tubulin expression), which shows an “accelerated” neurogenic progress with respect to the controls. The results also suggest that A-CSF could have an inhibitory effect on neurogenesis from both the quantitative (reducing the number of new neurons) and qualitative (delaying the transition from newborn to young neuron) aspects. In our study, we used coronal sections of adult mouse brain; therefore, we were unable to track the final destination of these migratory neuroblasts (olfactory bulb). However, our data relative to Doublecortin positive cells, strongly suggest that E-CSF’s influence on NSC activity involves also an increase in normal migratory behavior. Finally, our study suggests that the final destination of the new neurons generated under the E-CSF influence in the SVZ could be transformation into mature neurons. Taking together the results reported here, E-CSF seems to activate the main steps of SVZ NSCs behavior, increasing neurogenesis.

Here we show that the neurogenic instructive signals present in E-CSF are capable of increasing the physiological activity of adult brain NSCs. The CSF is deemed a key factor in contributing to the niche microenvironment diffusible signals in the developing brain (Gato et al., 2005; Miyan et al., 2006; Chau et al., 2015), and in adult brain astrocytic precursors (Alvarez-Buylla and Lim, 2004). Previous studies have shown that E-CSF induces in embryonic NSCs niche cell survival, replication and neuronal differentiation (Gato et al., 2005; Martin et al., 2009). Nevertheless, A-CSF does not maintain these neurogenic inductive properties in adult brain NSCs nor support neuron survival (Buddensiek et al., 2010; Carnicero et al., 2013; Ma et al., 2013). These studies confirm an ontogenic loss of the neurogenic inductive properties of CSF, which can be explained by the progressive modification of the proteic composition of this fluid across life revealed by proteomic studies (Parada et al., 2005a, 2006; Zappaterra et al., 2007). These changes could be an ontogenic adaptation to specific necessities to generate new neurons.

Our results show that the local application of a neurogenic stimulus (such as E-CSF) to the adult SVZ niche brings about complete activation of the NSCs including the two main steps in neurogenesis activity: an increase in mitotic activity leading to precursor population expansion and subsequent differentiation to young neurons able to migrate and progress to a mature state. This is in agreement with previous research showing the E-CSF neurogenic properties at the earliest stages of brain development (Zappaterra and Lehtinen, 2012; Gato et al., 2014), and also with the report of changes in the properties in A-CSF, described as mitogenic but “non” neurogenic in the adult brain neurogenic niche (Buddensiek et al., 2010; Carnicero et al., 2013) and in the embryonic brain (Gato and Desmond, 2009). Other specific properties of A-CSF in the neurogenic niche include migratory guidance instructive signals for reaching the proper destination (Sawamoto et al., 2006).

Taking into account these data together with the cellular lineage of the NSCs concept, we propose the theory that CSF is a changing brain component which evolves throughout life and plays a key role in neurogenesis.

Further studies are necessary to better understand the molecular basis of embryonic CSF neurogenic properties and the changes induced by age, which probably limit adult neuroregeneration.

About how E-CSF influences NSC behavior, E-CSF is a complex fluid involved in neurogenesis (Gato et al., 2005; Parada et al., 2005b; Martin et al., 2009; Desmond et al., 2014; Chau et al., 2015). Studies conducted to identify active components in CSF as growth and transcriptional factors have been developed and shown the implication of FGF2, IGF1 and EGF (among others) in their mitogenic properties (Miyan et al., 2003; Martín et al., 2006; Lathia et al., 2007; Zappaterra and Lehtinen, 2012). In addition, the influence of E-CSF on neurogenesis have been related with the retinol binding protein, and their involvement in Retinoic Acid synthesis (Parada et al., 2008; Alonso et al., 2011, 2014; Chang et al., 2016).

### Concluding Remarks

This study supports the idea that the CSF could actually be considered an “inner lake” for brain cellular intercommunication, playing a key role in establishing the niche microenvironment in the developing and adult brain.

Here we report new data relating to E-CSF properties. This represents the ability to induce global changes in adult mouse SVZ niche NSCs activity, promoting an expansive increase in the NSCs population, migratory activity and neurogenesis with neuronal maturation.

Our main conclusion is that E-CSF could be a cocktail of instructive signals, with hidden key information on neurogenesis control from NSCs throughout life. Further studies are necessary to check the usefulness of these properties in adult brain “*in vivo*” neurogenesis induction.

## Author Contributions

MIA, AG and FL designed and performed most of the experiments, analyzed data, prepared all figures and wrote the manuscript. EC, JAM contributed to the experiments. AM and JMFF collected the mouse embryonic fluid. MED helped to write the manuscript.

## Conflict of Interest Statement

The authors declare that the research was conducted in the absence of any commercial or financial relationships that could be construed as a potential conflict of interest.
